# Identification of phytoplankton isolates from the eastern Canadian waters using long-read sequencing

**DOI:** 10.1093/plankt/fbae043

**Published:** 2024-10-03

**Authors:** Solenn Mordret, Jenna MacKinnon, Joerg Behnke, Stephen J B O’Leary, Caroline Chénard

**Affiliations:** Aquatic and Crop Resource Development-National Research Council Canada, 1411 Oxford Street, Halifax, Nova Scotia B3H 3Z1, Canada; Aquatic and Crop Resource Development-National Research Council Canada, 1411 Oxford Street, Halifax, Nova Scotia B3H 3Z1, Canada; Aquatic and Crop Resource Development-National Research Council Canada, 1411 Oxford Street, Halifax, Nova Scotia B3H 3Z1, Canada; Aquatic and Crop Resource Development-National Research Council Canada, 1411 Oxford Street, Halifax, Nova Scotia B3H 3Z1, Canada; Aquatic and Crop Resource Development-National Research Council Canada, 1411 Oxford Street, Halifax, Nova Scotia B3H 3Z1, Canada

**Keywords:** culture collection, protists, microbial eukaryotes, picoeukaryotes, St. Lawrence estuary, Gulf of St. Lawrence, Scotian shelf, North Atlantic waters

## Abstract

As important primary producers and key components of biogeochemical processes, phytoplankton communities are highly diverse and continually adapt to changes in the environment, impacting the entire marine ecosystem. Consequently, it remains important to isolate, culture and properly describe new phytoplankton strains to provide relevant model organisms for laboratory research and accurate reference sequences for identification. Here, 73 phytoplankton strains from the eastern Canadian waters were isolated and genetically characterized using a long rRNA fragment (~4000 bp) covering the 18S, ITS and 28S rRNA regions generated with long-read sequencing technology. While most strains (66%) were accurately identified using the partial 18S rRNA gene (~1200 bp—V4–V9), this study broadens the current 28S rRNA reference database by incorporating 41 distinct strains including 30 unique species. In addition, previously unpublished long-read reference sequences were generated for a few genera, including *Picochlorum and Droopiella.* Species that were previously poorly described in the eastern Canadian waters were also genetically characterized, including a *Chaetoceros similis* strain. Overall, this work expands the total number of long-read references, an essential resource for accurate identification of phytoplankton and environmental sequences, thereby advancing the taxonomic resolution that could lead to a better understanding of environmental microbial diversity.

## INTRODUCTION

Phytoplankton account for approximately 50% of global primary production and play a pivotal role in shaping marine ecosystems. Their direct impact as a nutrient source for higher trophic levels, including zooplankton and small fish, highlights their critical link in the transfer of energy and nutrients through marine food webs (Field *et al.*, 1998; Friend *et al.*, 2009). Additionally, phytoplankton are crucial to marine biogeochemical processes, including the carbon cycle, where they remove CO_2_ from the atmosphere through photosynthesis and, upon decomposition, sequester carbon in the form of organic matter in the deep ocean (Falkowski, 1994; Falkowski, 2012).

The eastern Canadian waters includes a network of fresh, brackish and marine environments with the St. Lawrence Estuary, the Gulf of St. Lawrence, the Scotian Shelf and the Newfoundland Shelf. In those waters, phytoplankton are a key component of the regional dynamics with the advent of biannual (spring and autumn) blooms driven by environmental factors such as sea surface temperature (Li *et al.*, 2006; Zhi-Ping *et al.*, 2010; Barton *et al.*, 2015; Friedland *et al.*, 2016) and wind (Péquin *et al.*, 2017). In addition to high productivity, the eastern Canadian waters have been recognized as a region of high phytoplankton diversity and dynamic community composition using molecular work (Dasilva *et al.*, 2014; Robicheau *et al.*, 2022). However, few recent studies have focused on the genetic characterization of phytoplankton strains that originate from this area. This highlights the importance of isolation and culturing to provide accurate reference sequences and new model species for a better understanding of the biology, ecology and evolution of local phytoplankton.

The most common molecular method used to identify phytoplankton species consists of using the ribosomal ribonucleic acid (rRNA) genetic information, as rRNA sequences include both highly conserved and highly variable regions (Pawlowski *et al.*, 2012; Guillou *et al.*, 2013; del Campo *et al.*, 2018; Santoferrara *et al.*, 2020; Burki *et al.*, 2021; Latz *et al.*, 2022). Variable regions of the 18S rRNA or 28S rRNA are the target regions from the rRNA sequences (Lopes Dos Santos *et al.*, 2022). Sanger sequencing has been extensively used to generate prokaryotic and eukaryotic reference sequences as it is a reliable and cost-efficient method. In the recent years, with the advent of high-throughput sequencing platforms, phytoplankton community analysis has generally been focused on sequencing short 18S, 28S or ITS rRNA fragments between 150 and 400 base pairs (bp) (Latz *et al.*, 2022). These short reads pose limitations on the taxonomic resolution of sequence data, influencing the phylogenetic analysis, which sometimes results in insufficient information for species-level characterization of microbial eukaryotes (Maloney *et al.*, 2023; Ohta *et al.*, 2023). In addition, a good assignment and identification of sequencing data crucially depends on the quality of the reference database used.

With the emergence of long-read sequencing technologies, such as Pacific Bioscience (PacBio) or Oxford Nanopore Technology™, there is growing interest in targeted long-read sequencing covering a large portion of the rRNA genes to improve taxonomic resolution (Heeger *et al.*, 2018; Angell *et al.*, 2020; Jamy *et al.*, 2020; Tedersoo *et al.*, 2021). For prokaryotes, a sequence of approximately 1500 bp within the 16S rRNA gene is widely used (Ciuffreda *et al.*, 2021) to identify isolates (Johnson *et al.*, 2019) and decipher community structures (Matsuo *et al.*, 2021; Santos *et al.*, 2020; Urban *et al.*, 2021). Other studies have demonstrated the advantage of sequencing the full-length ribosomal operon for an accurate identification of prokaryotic communities (Cuscó *et al.*, 2018; Martijn *et al.*, 2019). Despite the advantages demonstrated for prokaryotes, relatively few studies have focused on using long-read sequencing to capture the genetic diversity of microbial eukaryotes (Heeger *et al.*, 2018; Davidov *et al.*, 2020; Jamy *et al.*, 2020). Among those studies, the majority used long-read sequencing for the identification of fungal communities (D’Andreano *et al.*, 2021; Ohta *et al.*, 2023; Yu *et al.*, 2023) or eukaryotic gut microbiota (Popovic *et al.*, 2018; Maloney *et al.*, 2021; Lu *et al.*, 2022). Few studies have focused on phytoplankton communities (Hatfield *et al.*, 2020; Jamy *et al.*, 2020; Gaonkar and Campbell, 2024). Hatfield and colleagues (Hatfield *et al.*, 2020) used Oxford Nanopore Technology for rapid discrimination of potentially harmful dinoflagellates, focusing on the genus *Alexandrium*, while Jamy and colleagues (Jamy *et al.*, 2020) introduced long-read ribosomal sequencing with PacBio as a method to phylogenetically and taxonomically resolve environmental diversity. The utilization of long-read sequencing technologies, especially in the exploration of microbial eukaryotes, remains a promising avenue for enhancing taxonomic resolution and significantly contributing to the understanding of microbial diversity.

Generating long reads expedites the production of more highly informative reference sequences. Combining the information for 18S rRNA and 28S rRNA often resolves phylogenetic relationships between closely related species (Santoferrara *et al.*, 2012; Pochon *et al.*, 2014). In a similar fashion, multigene phylogenies are now commonly implemented to resolve the molecular taxonomy of unicellular eukaryotes (Caron, 2013; Santoferrara *et al.*, 2016; Keeling and del Campo, 2017). Integrating long reads accelerates the process of obtaining sufficient genetic information to enhance the precision of phylogenetic analyses and contributes to a more nuanced understanding of genetic relationships within biological taxa.

The aim of this study was to apply long-read sequencing technology (Oxford Nanopore Technologies) to target the rRNA fragment that includes the 18S (V4–V9 regions), ITS1, 5.8S, ITS2 and 28S (D1–D8 domains) marker regions (hereafter 18S-ITS-28S) from 73 phytoplankton strains, which were recently isolated from different regions of the eastern Canadian waters. In addition to adding new reference sequences of the full 18S-ITS-28S fragment of the rRNA genes for a range of phytoplankton groups, this study provides previously unpublished 28S rRNA reference sequences for 41 different strains including 30 unique species. Those sequences also originate from strains that were previously undescribed and/or poorly characterized in Canadian waters. Overall, these additions to the long-read references will improve the taxonomic identification of future isolates and support the analysis of environmental phytoplankton sequences.

## METHOD

### Sampling, isolation, and culturing

**Fig. 1:**
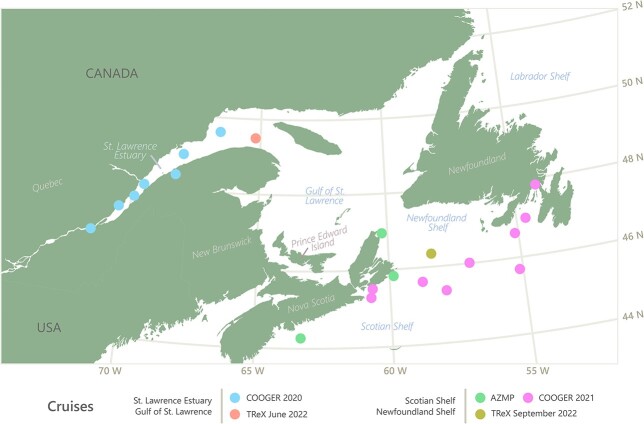
Map of sampling locations from which strains were isolated during the different cruises (AMZP, COOGER 2020, COOGER 2021, TReX June 2022 and TReX September 2022) in the Canadian Northwest Atlantic between August 2020 and September 2022.

Phytoplankton single cells were isolated from natural seawater samples collected with a CTD-Rosette at the surface and at chlorophyll maximum depth during five research cruises over the Scotian Shelf, in the St. Lawrence Estuary, Gulf of St. Lawrence and on the Newfoundland Shelf (AZMP-2020, COOGER-2020, COOGER-2021, TrEX2-2022, TrEX3-2022) over a two-year period (Fig. 1, Table I). Phytoplanktons were isolated by dilution series, agar plating or single-cell isolation. Once a culture was established, strains were deposited in the Algal Genomics and Synthetic Biology (AGSB) culture collection maintained by the National Research Council of Canada in Halifax, Nova Scotia. Culture and LM pictures of isolates are available upon request to the corresponding author. Unialgal cultures from the AGSB culture collection are maintained at 8, 16 or 20°C on a 16:8 (light:dark) cycle under 30 μmol of light. Most cultures are maintained in a seawater L1 medium (NCMA/Bigelow Laboratory for Ocean Sciences, USA) and salinity of (~34 ppt) except for six strains AGSB-0011 (*Tetradesmus obliquus*), AGSB-0027 (*Discostella* sp.), AGSB-0030 (*T. obliquus*), AGSB-0040 (*Chlorella* sp.), AGSB-0041 (*Nitzschia* sp.) and AGSB-0102 (*Nitzschia* sp.) which are maintained in freshwater L1 medium.

### Nucleic acid extraction

Deoxyribonucleic acid (DNA) was extracted from unialgal cultures using the MasterPure™ Complete DNA and RNA Purification Kit (Epicenter Biotechnologies, USA) or DNeasy® Plant Pro Kit (Qiagen, USA) following the manufacturer’s instructions. A total of 1.5–10 mL of culture material was used for each DNA extraction, depending on the biomass of the culture. Genomic DNA was eluted in 25 μL of buffer TE for the MasterPure™ extractions or 75 μL of buffer EB for DNeasy® Plant Pro extractions.

### Gene amplification and sequencing

A long-read fragment covering a large part of the ribosomal genes was selected for amplification (~4200 bp) using a set of four primers (Supplementary Table I). The forward primers (SSU-F and 3NDF) used are located just before the 18S-V4 region and were combined with the reverse primer 21R, located just after the 28S-D3 domain (Supplementary Table I). When those sets of primers (SSU-F/3NDF associated with 21R) did not produce amplification, a shorter fragment was amplified using the set SSU-F and D3Ca-R primers, which cover the V4-18S rRNA to the D3-28S rRNA. Amplicons (18S-ITS-28S rRNA) were sequenced with Oxford Nanopore Technologies™ (ONT) using the Ligation Sequencing Kit (SQK-LKS109) and the PCR Barcoding Expansion 1–12 (EXP-PBC001) on a Flongle flow cell (R9.4.1). PCR amplification and sequencing were performed following the protocol from Egeter *et al.*, 2022. All PCR reactions were carried out using LongAmp® Taq 2x Master Mix (New England Biolabs, USA) following the manufacturers’ instructions for enzyme and primers’ concentrations. A first PCR reaction was set up with primers that include MinION adapters (5′-TTT CTG TTG GTG CTG ATA TTG C-forward primer-3′, 5´-ACT TGC CTG TCG CTC TAT CTT C-reverse primer-3′) (Supplementary Table I), with the following conditions: an initial denaturation step of 30 s at 94°C, followed by 30 cycles of 10 s of denaturation at 94°C, 30 s of annealing at 60°C and 2 min 30 s of elongation at 65°C, then a final step of elongation at 65°C for 10 min.

**Table I:** Description of all AGSB strains isolated, cultured and sequenced for this study

Strains IDs				Sampling					Culturing	
Accession	Strains	Species	Division	Date	Depth (m)	Latitude	Longitude	Cruise	Temp. (°C)	Marine/Freshwater
OR922658	AGSB-0001	*Nitzschia* sp.	Stramenopiles	21 August 2020	2	47.71833 N	69.88633 W	Coriolis 2020	8	Marine
OR922659	AGSB-0002	*Rhinomonas nottbecki*	Cryptophyta	20 August 2020	2	47.41567 N	70.44083 W	Coriolis 2020	8	Marine
OR922660	AGSB-0003	*Attheya septentrionalis*	Stramenopiles	22 August 2020	2	48.05767 N	69.54883 W	Coriolis 2020	8	Marine
OR922661	AGSB-0004	**Attheya* septentrionalis*	Stramenopiles	22 August 2020	2	48.05767 N	69.54883 W	Coriolis 2020	8	Marine
OR922662	AGSB-0005	*Attheya septentrionalis*	Stramenopiles	27 August 2020	2	48.954 N	68.0945 W	Coriolis 2020	8	Marine
OR922663	AGSB-0006	*Rhinomonas nottbecki*	Cryptophyta	29 August 2020	2	49.616 N	66.66117 W	Coriolis 2020	8	Marine
OR922664	AGSB-0011	*Tetradesmus obliquus*	Chlorophyta	19 August 2020	2	46.7155 N	71.39167 W	Coriolis 2020	20	Freshwater
OR922665	AGSB-0012	*Picochlorum* sp.	Chlorophyta	01 August 2021	20	47.828283 N	54.07993 W	COOGER 2021	16	Marine
OR922666	AGSB-0013	*Prorocentrum pervagatum*	Alveolata	01 August 2021	20	47.828283 N	54.07993 W	COOGER 2021	16	Marine
OR922667	AGSB-0014	*Pyramimonas* sp.	Chlorophyta	01 August 2021	20	47.828283 N	54.07993 W	COOGER 2021	16	Marine
OR922668	AGSB-0015	*Chaetoceros socialis*	Stramenopiles	08 August 2021	28	45.510471 N	60.65152 W	COOGER 2021	16	Marine
OR922669	AGSB-0016	*Nephroselmis pyriformis*	Chlorophyta	08 August 2021	28	45.510471 N	60.65152 W	COOGER 2021	16	Marine
OR922670	AGSB-0017	*Mediolabrus comicus*	Stramenopiles	08 August 2021	28	45.510471 N	60.65152 W	COOGER 2021	16	Marine
OR922671	AGSB-0018	*Minidiscus trioculatus*	Stramenopiles	08 August 2021	28	45.510471 N	60.65152 W	COOGER 2021	16	Marine
OR922672	AGSB-0019	*Nitzschia* sp.	Stramenopiles	08 August 2021	28	45.510471 N	60.65152 W	COOGER 2021	16	Marine
OR922673	AGSB-0023	*Pyramimonas obovata*	Chlorophyta	04 August 2021	41	45.983369 N	57.516912 W	COOGER 2021	16	Marine
OR922674	AGSB-0025	*Minutocellus polymorphus*	Stramenopiles	08 August 2021	28	45.510471 N	60.65152 W	COOGER 2021	16	Marine
OR922675	AGSB-0026	*Dicrateria rotunda*	Haptophyta	26 August 2020	2	48.956739 N	68.085193 W	Coriolis 2020	8	Marine
OR922676	AGSB-0027	*Discostella* sp.	Stramenopiles	19 August 2020	3	46.7155 N	71.39167 W	Coriolis 2020	20	Freshwater
OR922677	AGSB-0030	*Tetradesmus obliquus*	Chlorophyta	19 August 2020	3	46.7155 N	71.39167 W	Coriolis 2020	20	Freshwater
OR922678	AGSB-0031	*Extubocellulus spinifer*	Stramenopiles	13 October 2020	1	46.9583 N	60.2167 W	AZMP	8	Marine
OR922679	AGSB-0032	**Attheya * septentrionalis*	Stramenopiles	13 October 2020	20	46.9583 N	60.2167 W	AZMP	8	Marine
OR922680	AGSB-0034	*Thalassiosira hispida*	Stramenopiles	09 October 2020	5	44.2667 N	63.3167 W	AZMP	8	Marine
OR922681	AGSB-0037	*Nannochloropsis granulata*	Stramenopiles	12 October 2020	1	45.825 N	59.85 W	AZMP	8	Marine
OR922682	AGSB-0038	*Dicrateria rotunda*	Haptophyta	12 October 2020	20	45.825 N	59.85 W	AZMP	8	Marine
OR922683	AGSB-0040	*Chlorella* sp.	Chlorophyta	19 October 2020	3	46.7155 N	71.39167 W	Coriolis 2020	20	Freshwater
OR922684	AGSB-0041	*Nitzschia* sp.	Stramenopiles	19 October 2020	3	46.7155 N	71.39167 W	Coriolis 2020	20	Freshwater
OR922685	AGSB-0044	*Chaetoceros tenuissimus*	Stramenopiles	04 August 2021	3	45.3573 N	57.91873 W	COOGER 2021	16	Marine
OR922686	AGSB-0045	**Chaetoceros *tenuissimus*	Stramenopiles	04 August 2021	3	45.3573 N	57.91873 W	COOGER 2021	16	Marine
OR922687	AGSB-0049	*Droopiella spaerckii*	Chlorophyta	04 August 2021	3	45.3573 N	57.91873 W	COOGER 2021	16	Marine
OR922688	AGSB-0052	**Chaetoceros *tenuissimus*	Stramenopiles	01 August 2020	2	47.109 N	54.479 W	COOGER 2021	16	Marine
OR922689	AGSB-0055	*Chlamydomonas* sp.	Chlorophyta	02 August 2021	2	46.998 N	54.6486 W	COOGER 2021	16	Marine
OR922690	AGSB-0057	*Thalassiosira aestivalis*	Stramenopiles	04 August 2021	47	46.0115 N	56.9749 W	COOGER 2021	16	Marine
OR922691	AGSB-0058	*Pyramimonas obovata*	Chlorophyta	04 August 2021	47	46.0115 N	56.9749 W	COOGER 2021	16	Marine
OR922692	AGSB-0061	*Heterocapsa steinii*	Alveolata	07 August 2021	2	45.62456 N	58.7791 W	COOGER 2021	16	Marine
OR922693	AGSB-0065	*Leptocylindrus minimus*	Stramenopiles	01 August 2021	20	47.828283 N	54.07993 W	COOGER 2021	16	Marine
OR922694	AGSB-0066	*Cylindrotheca closterium*	Stramenopiles	04 August 2021	41	45.983369 N	57.516912 W	COOGER 2021	16	Marine
OR922695	AGSB-0067	*Skeletonema marinoi*	Stramenopiles	04 August 2021	41	45.983369 N	57.516912 W	COOGER 2021	16	Marine
OR922696	AGSB-0068	*Skeletonema marinoi*	Stramenopiles	08 August 2021	28	45.510471 N	60.65152 W	COOGER 2021	16	Marine
OR922697	AGSB-0071	**Chaetoceros* socialis*	Stramenopiles	08 August 2021	28	45.510471 N	60.65152 W	COOGER 2021	16	Marine
OR922698	AGSB-0082	**Chaetoceros *tenuissimus*	Stramenopiles	04 August 2021	2	45.3573 N	57.91873 W	COOGER 2021	16	Marine
OR922699	AGSB-0085	**Chaetoceros *tenuissimus*	Stramenopiles	03 August 2021	25	46.6398 N	55.1322 W	COOGER 2021	16	Marine
OR922700	AGSB-0087	**Chaetoceros* tenuissimus*	Stramenopiles	02 August 2021	2	46.998 N	54.6486 W	COOGER 2021	16	Marine
OR922701	AGSB-0088	*Dicrateria rotunda*	Haptophyta	04 August 2021	28	45.983369 N	57.516912 W	COOGER 2021	16	Marine
OR922702	AGSB-0089	*Chlamydomonas* sp.	Chlorophyta	13 October 2020	1	46.9583 N	60.2167 W	AZMP	8	Marine
OR922703	AGSB-0090	*Extubocellulus spinifer*	Stramenopiles	13 October 2020	20	46.9583 N	60.2167 W	AZMP	8	Marine
OR922704	AGSB-0094	*Chaetoceros neogracilis*	Stramenopiles	09 October 2020	20	42.8317 N	61.7333 W	AZMP	8	Marine
OR922705	AGSB-0096	*Skeletonema marinoi*	Stramenopiles	22 August 2020	2	48.05767 N	69.54883 W	Coriolis 2020	8	Marine
OR922706	AGSB-0097	*Nephroselmis pyriformis*	Chlorophyta	29 August 2020	2	49.616 N	66.66117 W	Coriolis 2020	8	Marine
OR922707	AGSB-0102	*Nitzschia* sp.	Stramenopiles	19 August 2020	3	46.7155 N	71.39167 W	Coriolis 2020	20	Freshwater
OR922708	AGSB-0111	**Thalassiosira* aestivalis*	Stramenopiles	03 August 2021	25	46.6398 N	55.1322 W	COOGER 2021	16	Marine
OR922709	AGSB-0114	*Chaetoceros similis*	Stramenopiles	04 August 2021	47	46.0115 N	56.9749 W	COOGER 2021	16	Marine
OR922710	AGSB-0118	*Picochlorum* sp.	Chlorophyta	03 August 2021	25	46.6398 N	55.1322 W	COOGER 2021	16	Marine
OR922711	AGSB-0122	*Chlamydomonas* sp.	Chlorophyta	08 August 2021	2	45.69166 N	55.13227 W	COOGER 2021	16	Marine
OR922712	AGSB-0123	**Chaetoceros* tenuissimus*	Stramenopiles	08 August 2021	2	45.510471 N	60.65152 W	COOGER 2021	16	Marine
OR922713	AGSB-0127	*Dicrateria rotunda*	Haptophyta	04 August 2021	41	45.983369 N	57.516912 W	COOGER 2021	16	Marine
OR922714	AGSB-0128	*Picochlorum* sp	Chlorophyta	04 August 2021	41	45.983369 N	57.516912 W	COOGER 2021	16	Marine
OR922715	AGSB-0129	**Thalassiosira* aestivalis*	Stramenopiles	03 August 2021	25	46.6398 N	55.1322 W	COOGER 2021	16	Marine
OR922716	AGSB-0130	*Picochlorum* sp.	Chlorophyta	04 August 2021	41	45.983369 N	57.516912 W	COOGER 2021	16	Marine
OR922717	AGSB-0133	**Cylindrotheca *closterium*	Stramenopiles	01 August 2021	20	47.828283 N	54.07993 W	COOGER 2021	16	Marine
OR922718	AGSB-0137	**Chaetoceros* tenuissimus*	Stramenopiles	01 August 2021	2	47.109 N	54.479 W	COOGER 2021	16	Marine
OR922719	AGSB-0168	*Thalassiosira pseudonana*	Stramenopiles	20 August 2020	2	48.66683 N	68.58117 W	Coriolis 2020	16	Marine
OR922720	AGSB-0176	**Chaetoceros* tenuissimus*	Stramenopiles	02 August 2021	2	46.998 N	54.6486 W	COOGER 2021	16	Marine
OR922721	AGSB-0181	*Fragilaria famelica*	Stramenopiles	08 August 2021	23	45.28369 N	60.71225 W	COOGER 2021	16	Marine
OR922722	AGSB-0186	*Chaetoceros* sp.	Stramenopiles	12 August 2021	3	42.402761 N	61.051786 W	COOGER 2021	16	Marine
OR922723	AGSB-0188	**Thalassiosira* pseudonana*	Stramenopiles	10 August 2021	3	45.467761 N	61.118908 W	COOGER 2021	16	Marine
OR922724	AGSB-0210	*Picochlorum* sp.	Chlorophyta	02 August 2021	2	46.998 N	54.6486 W	COOGER 2021	16	Marine
OR922725	AGSB-0221	*Actinocyclus* sp.	Stramenopiles	13 June 2022	5	49.4927 N	65.208 W	Trex June 2022	8	Marine
OR922726	AGSB-0236	*Droopiella spaerckii*	Chlorophyta	15 September 2022	20	46.3419 N	58.3811 W	TReX September 2022	16	Marine
OR922727	AGSB-0242	*Pyramimomas propulsa*	Chlorophyta	15 September 2022	20	46.3419 N	58.3811 W	TReX September 2022	16	Marine
OR922728	AGSB-0244	* Pyramimonas obovata*	Chlorophyta	15 September 2022	20	46.3419 N	58.3811 W	TReX September 2022	16	Marine
OR922729	AGSB-0253	*Picochlorum* sp.	Chlorophyta	15 September 2022	20	46.3419 N	58.3811 W	TReX September 2022	16	Marine
OR922730	AGSB-0254	*Mamiella gilva*	Chlorophyta	15 September 2022	20	46.3419 N	58.3811 W	TReX September 2022	16	Marine

The AGSB strains used in this study were collected during various cruises in the Canadian Northwest Atlantic between August 2020 and September 2022.

The PCR reactions were purified using a 0.45x volume of AMPure XP beads (Beckman Coulter, USA), incubated on a HulaMixer™ at room temperature for 5 min, then washed twice with 200 μL fresh 70% ethanol. The beads were allowed to dry for 2 min before DNA elution in 45 μL of nuclease-free water. To allow the pooling and sequencing of multiple sequencing libraries, a second PCR amplification (PCR barcoding) was performed using 100–200 fmol DNA from the first round PCR and PCR Barcodes (EXP-PBC001) with the following conditions: an initial denaturation step of 3 min at 95°C, followed by 30 cycles of 10 s of denaturation at 95°C, 30 s of annealing at 62°C and 4 min 30 s of elongation at 65°C, then a final step of elongation at 65°C for 10 min.

After amplification, the barcoded PCR reactions were purified using a 0.4x volume of AMPure XP beads, incubated on a HulaMixer™ at room temperature for 5 min, washed twice with 200 μL fresh 70% ethanol. The beads were allowed to dry for 2 min before DNA elution in 15 μL of nuclease-free water. Each purified PCR product was quantified with the Qubit™ dsDNA BR Assay (Thermo Fisher Scientific) using the manufacturer’s protocol after which ~ 100 ng of DNA from each sample was pooled into a single combined aliquot for sequencing with a total volume of 23.5 μL.

The remaining steps were performed according to the Nanopore protocol (Genomic DNA by Ligation-SQK-LSK109, Version: GDE_9063_v109_revAE_14Aug2019). For each Flongle cell, 12 different isolates were pooled together and sequenced on a MinIT device (MinIT Release 21.11.7) for 24 hrs. Between 17.41 and 219.43 k, reads were generated during each run.

### Bioinformatics analysis

Basecalling of the Fast5 files were performed with the MinIT device, while demultiplexing and adapter trimming were completed using Porechop (v. 0.2.4 [Wick *et al.*, 2017]). Nanofilt was used to remove reads below 2800 bp and above 6000 bp (De Coster *et al.*, 2018) with the exception of sequences obtained for AGSB-0031 and AGSB-0090 isolates, which included numerous inserts of 150 to 1100 bp in the 18S rRNA and for which Nanofilt length selection was made for sequences between 5000 and 10 000 bp. Chimeras were removed using yacrd and minimap2 (Li, 2021). The retained reads were clustered into a consensus sequence using isONclust (Sahlin and Medvedev, 2019) and aligned using MAFFT (Katoh *et al.*, 2002; Katoh and Standley, 2013). Low-quality sections at the edges of the sequences were removed. Sequences were also inspected and manually edited to correct for homopolymers using a multisequences alignment with MAFFT for each of the taxonomic groups (i.e. stramenopiles, chlorophytes, haptoptophytes, etc.), which included our sequence and from closely related reference sequences. The full consensus sequences produced in this study were first aligned with MAFFT and primers (i.e. 18S-V9 reverse primer “1510F” and 28S forward primer “D1R-C_m”—see Supplementary Table II) were detected *in silico* with a search tool on Geneious Prime. The 18S and 28S fragments were then extracted separately and saved in different files.

### Molecular taxonomic identification

Molecular taxonomic identification for each culture was performed via Basic Local Alignment Search Tool (BLAST) (Camacho *et al.*, 2009) in June 2023, using the following markers: 18S rRNA, 28S rRNA sequences and 18S-ITS-28S fragments. Species identification for each culture was first assessed via BLAST (Blastn) on the NCBI reference database excluding uncultured strains. Top BLAST results for individual 18S and 28S sequences were sorted by percentage of identity to find the best match with shorter reference sequence, while 18S-ITS-28S BLAST results were sorted by E-value. In case of identification dissimilarity between the different markers, the taxonomy resolved from the BLAST with the 18S markers was favored, unless 28S similarity was higher than 18S. All uncultured and environmental sample sequences were excluded from the BLAST for molecular taxonomic identification.

Taxonomical nomenclature was further verified using both AlgaeBase (Guiry and Guiry, 2024) and Protist Ribosomal Reference database (PR^2^) (Guillou *et al.*, 2013). While the AlgaeBase was used for species classification, the PR^2^ was used as a reference for schematic systems of identified strains. Isolates with a best BLAST result similarity (percentage of identity) higher than 99% for the 18S or the 28S rRNA were assigned at species level, unless best BLAST result reference was not assigned at species level. Other isolates were assigned at genus level with best BLAST results within 97% similarity in the 28S.

### Phylogenetic analysis

Phylogenetic trees were built using the sequenced strains and references sequences. Sequences covering the ribosomal 18S-ITS-28S fragment for both Bacillariophyta and Chlorophyta greater than 3500 bp were downloaded from Genbank (July 2023) and trees were generated. Genbank sequences that did not align or cover the entire length of query sequences were removed. Only reference sequences assigned at least to the genus level or higher and with a good nucleotide quality (<20 ambiguities) were retained. Multiple alignments with query and Genbank sequences were performed using MAFFT (v. 7.490) implemented in Geneious Prime (v. 2023.2.1). Phylogenies were generated using FastTree (v. 2.1.11; (Price *et al.*, 2010)) and used to verify all sequences. Based on those phylogenies, redundant species were removed from the phylogenetic analysis. Similarly only one representative sequence per species generated in this study was added to the alignment. Two sequences from the Eustigmatophyceae, *Nannochloropsis gaditana* (AZIL01002195) and *N. granulata* (AGSB-0037) were used as outgroups for the Bacillariophyta, while *Verdigellas peltata* (LT174528) was chosen to root the Chlorophyta phylogeny. Final multiple alignments of a genomic fragment that includes 18S-ITS1–5.8S-ITS2-28S rRNA region were composed of 72 sequences (including 25 AGSB sequences for phylogeny Fig. 4) for the Bacillariophyta and 34 sequences (including 14 AGSB sequences for phylogeny Fig. 5) for the Chlorophyta. Total length of matrices was 8638 and 6581 nucleotides for the Bacillariophyta and the Chlorophyta, respectively. Phylogenies were inferred using both MrBayes (v.3.2.6, Ronquist and Huelsenbeck, 2003) and RAxML (v. 8.2.11, Stamatakis, 2014) plugins as implemented in Geneious Prime. Bayesian inference analyses were performed with the following parameters: 1 100 000 generations per run, four independent runs, 100 000 burn-in length and 200 subsampling frequency. Robustness of inferred topologies was also assessed by running 1000 bootstraps for RAxML. For both Bayesian and RAxML analyses, the matrices were partitioned in three different sections representing (i) the 18S rRNA (V4-V9), (ii) the ITS (ITS1–5.8S-ITS2) and (iii) the 28S rRNA (D1-D8) using universal 18S reverse and 28S forward primers (Supplementary Table II). The general time-reversible model with gamma distribution of rate variation (GTR + GAMMA) was selected based on JmodelTest (Darriba *et al.*, 2012) for each partition matrix. Bayesian trees were first edited in FigTree (v. 1.4.4), and corresponding RAxML boostraps were added to the trees using Inkscape (v. 1.1.2).

### Accession numbers

The rRNA genes sequences generated in this study were deposited in NCBI under the following accession numbers: OR922658 to OR922730.

## RESULTS

### Quality of sequencing results

**Fig. 2:**
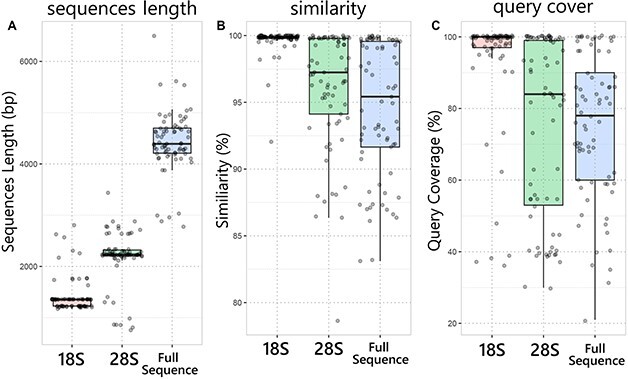
Boxplots with jittered points summarizing BLAST results in terms of (**a**) sequence length for each marker (18S, 28S rRNA and full sequence in bp) (**b**) similarity (sort by percentage identity %) compared to the best reference, and (**c**) query coverage (sort by query coverage in %) compared to the best reference.

The length of the rRNA fragments amplified and sequenced ranged from 2775 to 6497 bp. The median for the sequence length was 1355 bp for the 18S rRNA, 2234 bp for the 28S rRNA and 4391 bp for the full-length sequence (18S-ITS-28S fragment) (Fig. 2A, Supplementary Table III). The sequence length varied due to the use of two different primer sets and the presence of long inserts in the 18S or 28S region of some isolates. For example, the isolate AGSB-0031 (*Extubocellulus spinifer*) generated a sequence of 6497 bp, which included four large inserts (>100 bp) in its 18S rRNA gene, while the isolate AGSB-0055 (*Chlamydomonas* sp.) generated a sequence of 4540 bp as a result of a 429 bp insert in the 28S rRNA.

Taxonomic molecular identification for each culture via BLAST (Camacho *et al.*, 2009) demonstrated that the percent similarity was generally higher for the 18S marker than the 28S marker and the full sequence (18S-ITS-28S fragment) (Fig. 2B, Supplementary Table III). The median for percent similarity was 99.89% for the 18S rRNA, 97.24% for the 28S rRNA and 95.42% for the full sequence of the 18S-ITS-28S fragment. The long-read reference sequence compared to the shorter query might have influenced those results. There were some exceptions, including the strain AGSB-0027 *(Discotella* sp.) demonstrating a 92.02% similarity to *Discotella* sp. (DQ514902) as the closest hit for the 18S marker and a closest hit with a 97.25% similarity to *D. nipponica (*AB831889) for the 28S marker (Supplementary Table III). The strain AGSB-0082 (*Chaetoceros tenuissimus)* demonstrated a closest hit with a 96.28% similarity to *C. dayaensis* (KM401854) for the 18S marker and a closest hit with a 99.83% similarity to *C. tenuissimus* (MK193876) for the 28S marker (Supplementary Table III).

BLAST analysis also demonstrated that the query coverage was generally higher for the 18S marker than the 28S marker and the full sequence (18S-ITS-28S fragment). The median for the percent query coverage was 100% for the 18S rRNA, 84% for the 28S rRNA and 78% for the full-length sequence (18S-ITS-28S fragment) (Fig. 2C, Supplementary Table III). The 28S rRNA marker exhibited the largest variability in percent query coverage ranging from 30 to 100%.

### Diversity of the characterized cultures

**Fig. 3:**
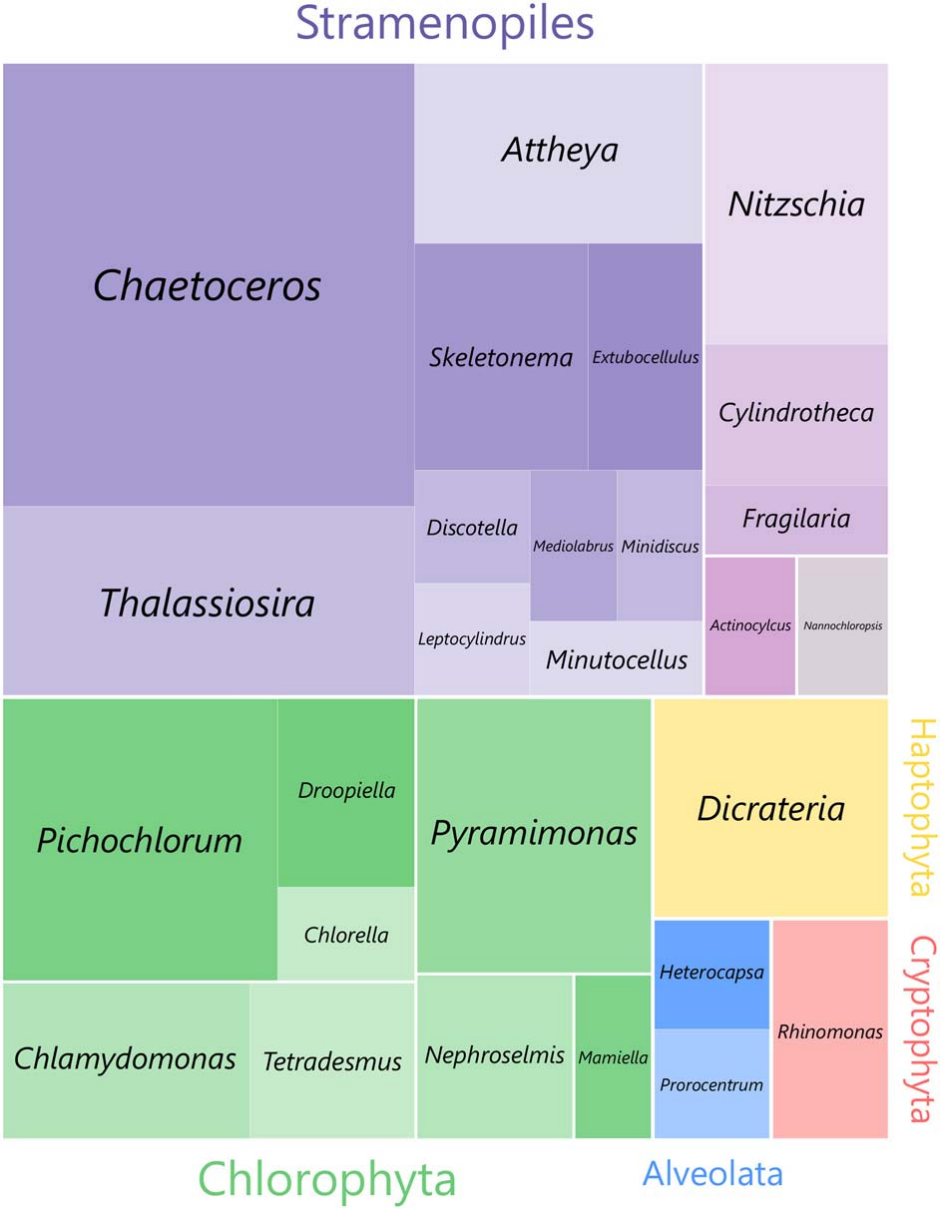
Treemap showing overall diversity at the division and genus levels for the 73 AGSB strains isolated, cultured and sequenced for this study.

Unialgal cultures originated from samples collected at the surface (2 or 5 m) or at depth chlorophyll maximum (DCM) during the summer and autumn season (from June to October) (Table I) in the eastern Canadian waters including the St. Lawrence Estuary, the Gulf of St. Lawrence, the Scotian Shelf and the Newfoundland Shelf (Table I, Fig. 1). Most strains were isolated from marine waters except for six strains: AGSB-0011 (*T. obliquus*), AGSB-0027 (*Discostella* sp.), AGSB-0030 (*T. obliquus*), AGSB-0040 (*Chlorella* sp.), AGSB-0041 (*Nitzschia* sp.) and AGSB-0102 (*Nitzschia* sp.). Those six strains were isolated from a station located west of Quebec City in the St. Lawrence fluvial estuary (i.e*.* freshwater estuary) (Fig. 1, Table I). A total of 73 isolates were genetically described using the long fragment of the ribosomal covering the 18S-ITS-28S rRNA marker genes. A total of 55 strains were identified at the species level (>99% similarity). Some strains with a high BLAST match were identified on genus level as the closest references were not identified at the species level. Those isolates grouped into 41 phylotypes from the Phyla Stramenopile, Chlorophyta, Haptophyta, Cryptophyta and Alveolata (Supplementary Table III, Fig. 3, Supplementary Fig. 1). The Phylum Stramenopile mostly included centric diatoms of the genera *Chaetoceros* (14 strains), *Thalassiosira* (6 strains), *Attheya* (4 strains) and *Skeletonema* (3 strains). Most pennate diatoms in the culture collection were from the genera *Nitzschia* (4 strains) and *Cylindrotheca* (2 strains). Stramenopiles also included one isolate belonging to the Eustigmatophyceae and the genus *Nannochloropsis*. The Phylum Chlorophyta mostly included members of the genera *Picochlorum* (6 strains), *Pyramimonas* (4 strains) and *Chlamydomonas* (3 strains). Of the 41 different phylotypes represented in this study, 27 of those were assigned at the species level based on high BLAST similarity results (>99.5% identity). The remaining 14 phylotypes were identified at the genus level using a combination of phylogenetic analyses supports and BLAST similarity results. The lack of species assignation for those phylotypes was mainly due to the absence of closely related references in the public database (NCBI). This is the case for AGSB-0114 (identified as *C. similis*) where the BLAST similarity results were insufficient for strain identification (91.65% for Best BLAST Identity) and neither 18S nor full 28S rRNA (KC986088, 564 bp) reference sequences were available for that strain. AGSB-0114 was identified more accurately using a combination of partial BLAST results and morphological characterization by and light microscopy (LM) (Supplementary Fig. 2) and scanning electron microscopy (SEM) (Supplementary Fig. 3) Combining the genetic and the morphological characterization demonstrated that AGSB-0114 should be assigned as *C.similis*.

Aveolates isolates include strains of *Prorocentrum pervagatum* (AGSB-0013) and *Heterocapsa steinii* (AGSB-0061), which were both identified with high confidence due to the high similarity to reference sequence (i.e. 100 and 99.93% identity with 18S rRNA reference, respectively). All strains of haptophytes were assigned to the same species, *Dicrateria rotundata*, matching with 99.94% similarity or higher with the 18S rRNA reference sequence. In the same way, the two Cryptophyte isolates collected were both assigned to the species *Rhinomonas nottbecki* following showing BLAST similarities of 99.92 and 99.84% for the 18S rRNA gene.

### Phylogenetic analysis

**Fig. 4:**
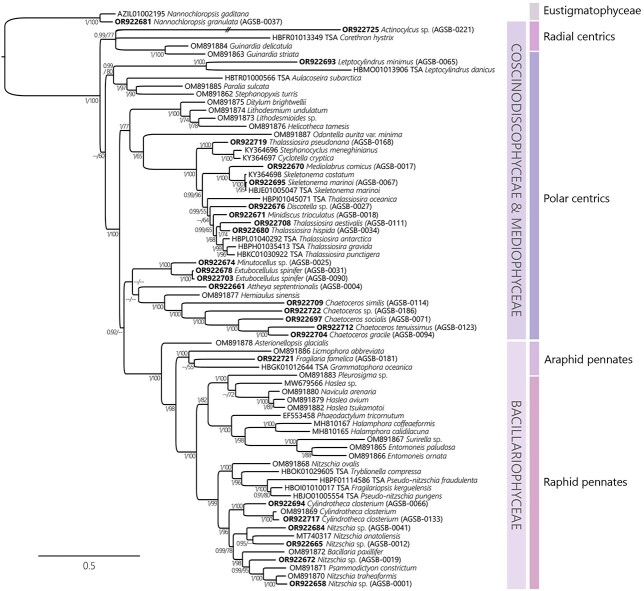
Phylogenies generated by the long reads using Bayesian concatenated tree showing the phylogenetic distribution of the Bacillariophyta based on the concatenated alignment (8638 bp matrix) of ribosomal genes 18S, 5.8S and 28S (including ITS1 and ITS2). The tree displays 25 AGSB strains (i.e. sequenced from this study) and 47 reference sequences from public repositories. Strains sequenced in this study are shown with the associated GenBank Accession Number in bold. Corresponding RAxML bootstraps were added to the tree at each node alongside Bayesian inferences. Bayesian support values and ML bootstraps under 0.90 and 50, respectively, are annotated as—long branch produced by *Actinocylcus* sp. was reduced by half (shown as//). For these phylogenies, Eustigmatophyceae sequences *N. gaditana* (AZIL01002195) and *N. granulata* (AGSB-0037) were used as outgroup. Informal clades as previously described by Theriot *et al.* (2010) are highlighted in the tree: Raphid pennates (which includes the class Bacillariophyceae), araphid pennates (which includes the class Fragilarophyceae) and polar and radial centrics (which includes the Coscinodiscophyceae and Mediophyceae).

**Fig. 5:**
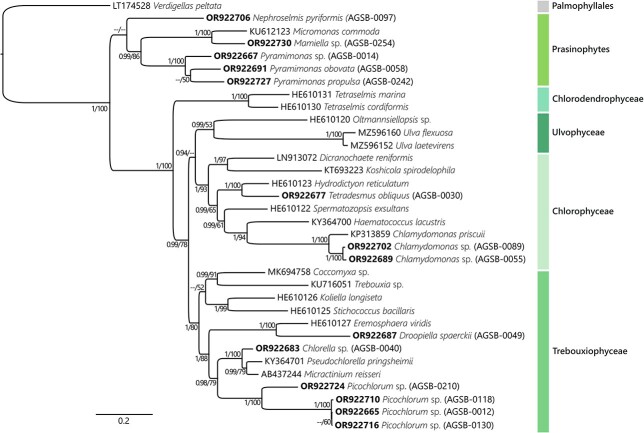
Phylogenies generated by the long reads using Bayesian concatenated tree showing the phylogenetic distribution of the Chlorophyta based on the concatenated alignment (6581 bp matrix) of ribosomal genes 18S, 5.8S and 28S (including ITS1 and ITS2). The tree displays 14 AGBS strains (i.e. sequenced in this study) and 20 reference sequences from public repositories. Strains sequenced in this study are shown with the associated GenBank Accession Number in bold. Corresponding RAxML bootstraps were added to the tree at each node alongside Bayesian inferences. Bayesian support values and ML bootstraps under 0.90 and 50, respectively, are annotated as—Palmophyllales sequence *V.peltata* (LT174528) was used as outgroup. The different clades highlight in the tree correspond to group at the class level as annotated in the PR^2^ database.

Multigene phylogenies using the rRNA fragment covering the 18S-ITS-28S region were performed for the Bacillariophyta (72 sequences including 25 AGSB sequences, Fig. 4) and the Chlorophyta (34 sequences including 14 AGSB sequences, Fig. 5). To target a greater diversity of genera and because of a limitation of available long-read sequences, the Bacillariophyta tree included 13 sequences from the transcriptome shotgun assembly (TSA) sequence database (https://www.ncbi.nlm.nih.gov/genbank/tsa/). In general, most strains from this study clustered with strong support at the genus level. For example, the diatom strain *Leptocylindrus minimus* (AGSB-0065) was most closely related to *L. danicus* (HBMO01013906 TSA) (Fig. 4). The two *Chlamydomonas* spp. (AGSB-0089, AGSB-0055) along with *C. priscuii* (KP313859) formed a monophyletic group (Fig. 5, posterior probabilities =1 and bootstrap support = 100%). Contrastingly, members of the genus *Thalassiosira*, did not form a monophyletic group and were found clustering with other genera of polar centric diatoms.

The phylogenetic analysis of the Bacillariophyta showed that the sequences scattered among the main clades of the tree with more occurrences in some groups rather than others (Fig. 4). The araphid pennates and the radial centrics were underrepresented among long-read references than the other groups. All pennate diatoms clustered within their respective groups, as shown with the strong support for the raphid pennates (posterior probabilities =1 and bootstrap support = 98%) and its sister clade the araphid pennates (posterior probabilities = 0.92, bootstrap support < 50%) (Fig. 4). Polar centric diatoms formed various clades representing the diverse families (Chaetocerotaceae, Cymatosiraceae, Thalassiosiraceae, Lithodesmiaceae, Skeletonemataceae and Stephanodiscaceae). Members of the family Thalassiosiraceae clustered as paraphyletic groups along with members of Skeletonemataceae and Stephanodiscaceae. The phylogenetic analysis resulted in monophyletic grouping of Cymatosiraceae and Chaetocerotaceae that only included long-read sequences generated in this study.

Less taxonomic groups within the Phylum Chlorophyta were represented by long-reads sequences. (Fig. 5). The tree topology distinctly showed the different families (Trebouxiophyceae, Chlorophyceae, Ulvophyceae, Chlorodendrophyceae and Prasinophytes) with strong support for most family’s clades. Sequences from this study only clustered among the following three families: Trebouxiophyceae (posterior probabilities =0.99 and bootstrap support = 78%), Chlorophyceae (posterior probabilities =0.94 and bootstrap support < 50%) and Prasinophytes (Posterior probabilities =1 and Bootstrap support = 100%). Within those families, this study provided the first long-read sequence references for several genera such as *Picochlorum* (AGSB-0118, AGSB-0012, AGSB-0130 and AGSB-0210) and *Droopiella* (AGSB-0049).

## DISCUSSION

### Long-read reference sequences

While short-read sequencing of taxonomically informative genes from organisms can quickly provide useful information for a general taxonomic description, long-read sequencing is more suitable to provide a more confident identification of discrete species. This is especially true for groups of organisms that are underrepresented in sequence databases or have poorly resolved assemblages. There is a lack of long-read sequences being deposited to reference databases. As a result, not all taxonomic groups are represented by long-read sequences and TSA sequences were included in the phylogenetic reconstruction of the Bacillariophyta tree to expand the diversity of the taxonomic groups targeted in the analysis.

This study demonstrated that the isolation and subsequent culturing of individual strains, followed by the sequencing of a long genomic fragment that includes 18S-ITS1-5.8S-ITS2-28S rRNA from major phytoplankton groups provided much needed reference sequences to improve the identification at species level. Combining 18S, ITS, and 28S rRNA in a single read using long-read sequencing improves species identification through integration of different rRNA markers used for species identification and phylogenetic analyses. In general, while many 18S rRNA sequences are available in full length in reference databases (Guillou *et al.*, 2013; del Campo *et al.*, 2018) most 28S rRNA sequences published in the reference databases only target a fragment of between 700 and 900 bp (Grzebyk *et al.*, 2022). However, 28S rRNA genes can include more variable regions, which may better resolve species level identification than the corresponding 18S rRNA fragment. While trying to detect marine harmful algal bloom species, Hatfield and co-authors (Hatfield *et al.*, 2020) did not take full advantage of the long-read capabilities as they only selected the 18S rRNA sequences for their analysis. Even if the 18S rRNA is more commonly used for taxonomic assignments, alternative gene markers such as the 28S rRNA and ITS regions are often essential for accurate identification (Pochon *et al.*, 2014; Grzebyk *et al.*, 2022). For example, *Chaetoceros neogracilis* isolates from the Beauford Sea shared identical 18S rRNA but represented four different clades based on the 28S rRNA and ITS phylogenies (Balzano *et al.*, 2017). The long-read sequences reported in this study helped to close the gap for some phytoplankton taxonomic groups where the characterization of the full rRNA has previously been overlooked. For example, this study provided the first long-read sequence references for the Chlorophyte genera *Picochlorum* (AGSB-0012, AGSB-0118, AGSB-0130 and AGSB-0210) and *Droopiella* (AGSB-0049).

In addition to adding long-read sequences to the reference databases for precise identification of strains, those sequences can further be used as references in multi-gene phylogenies. Multigene phylogenies increase the phylogenetic resolution in comparison with single-gene phylogenies such as the ones only including the 18S rRNA gene. Although the 18S rRNA gene possess the greatest number of references for microbial eukaryotes, the 18S rRNA alone has a limited phylogenetic resolution power. In fact, due to its limited number of phylogenetically informative sites, phylogenetic analyses using the 18S rRNA gene are prone to stochastic errors, which hamper the resolution of evolutionary relationships at deeper level between taxa (Martijn *et al.*, 2019). As 18S rRNA sequences contain fewer phylogenetically informative sites but more conserved regions, phylogenies based on this gene are often used to resolve relationships at higher phylogenetic levels (i.e. Order, Family or Genus level). Comparatively, the 28S rRNA gene often displays a deeper resolution power for numerous phytoplankton taxa (Bittner *et al.*, 2013; Grzebyk *et al.*, 2022) providing grounds for the description of numerous phytoplankton species. Aiming to increase the phylogenetic resolution, the concatenated phylogenies of the rRNA genes have been widely used for prokaryotes (Martijn *et al.*, 2019; D’Andreano *et al.*, 2021), but only for some eukaryotes (Santoferrara *et al.*, 2012; Orr *et al.*, 2018; Jamy *et al.*, 2020; Tillmann *et al.*, 2023). However, the entire rRNA genes sequences (e.g. 18S, ITS1, 5.8S, ITS2 and 28S rRNA) required for such phylogenetic analyses are generally not available for the majority of phytoplankton species. Indeed, most of the molecular taxonomic identification of individual species relies on sequencing techniques that only produce partial ribosomal information. Remarkably, the implementation of long-read sequencing technologies provides a convenient tool to sequence the large ribosomal fragment providing higher phylogenetic resolution in a very rapid and efficient way, and without having to sequence the full genome.

### Phylogenetic identification and taxonomic assignation of poorly characterized species

This work underlines the importance of still isolating, culturing and genetically characterizing new phytoplankton strains as it provides information on new species that could further be use for the biology, ecology and evolution of phytoplankton, both locally and globally. Several strains isolated in this study were previously reported as key members of phytoplankton communities on the Scotian Shelf (Dasilva *et al.*, 2014; Robicheau *et al.*, 2022), including *Leptocylindrus* (AGSB-0065), *Minidiscus trioculatus* (AGSB-0018), *Picochlorum spp.* (AGSB-0012, AGSB-0118, AGSB-0130 and AGSB-0210) and *Thalassiosira pseudonana* (AGSB-0168)*.*

Some of the strains isolated in this study were previously poorly characterized. For instance, the strain AGSB-0114, which was isolated from the DCM (47 m) in the Laurentian Channel, was identified as *C. similis* based on genetic and morphological characterization. From morphological data, *C. similis* is a well-described diatom found in temperate to cold waters and known to be prevalent in the North Atlantic Ocean (Hernández-Becerril, 2009; Hamsher *et al.*, 2013). The distinct morphological characteristics of *C. similis* include setae that arise from the valves at an angle of 30–35° with respect to the apical axis and exhibit slightly elongated vertically arranged poroids (as clearly visualize by SEM, see Supplementary Fig. 3). However, prior to this work, neither 18S rRNA nor the full 28S rRNA sequences were available for this species, making it difficult to taxonomically identify in environmental studies. Another *Chaetoceros* strain (AGSB-0186), isolated from surface waters on the Scotian Shelf, is also believed to be an undescribed species. Based on the BLAST similarity for the 18S rRNA, the strain AGSB-0186 was closely related to the *Chaetoceros* sp. strain RCC1810 (*C. pumilus/C. jonquieri* clade) (Campbell *et al.*, 2022) which was previously isolated from the Pacific Ocean waters near the French Polynesia. Despite having been isolated a few decades ago, *Chaetoceros* sp. strain RCC1810 (often referred as *Chaetoceros* sp. *jonquieri* or *C. jonquieri*) (Campbell *et al.*, 2022) has never been fully characterized. Reports of undescribed *Chaetoceros* species on the Scotian Shelf also suggested a novel genus group as environmental *Chaetoceros* sequences formed a distinct cluster with < 95% similarity to other known *Chaetoceros* species (Dasilva *et al.*, 2014). Previously, metabarcoding data from a time-series station in the coastal on the Scotian Shelf revealed a high relative abundance of *Chaetoceros,* especially in the spring (MacNeil *et al.*, 2022; Robicheau *et al.*, 2022). *Chaetoceros* is a highly diverse genus within the diatoms with more than 270 accepted species (Guiry and Guiry, 2024) and it is, therefore, not surprising to identify novel strains.

Similarly, *L. minimus* (AGSB-0065) was isolated from the coast of Newfoundland and was previously observed in Coastal Canadian Atlantic waters (Buxton and Brylinsky, 2005). Leptocylindroceae are known to have a strong seasonality in occurrence based on temperature and day-length (Nanjappa *et al.*, 2013, 2014). Those sequences supported the hypothesis that *L. minimus* (AGSB-0065) can be found in colder waters as reported in previous studies from the European coastal regions (Roscoff), Oslo Fjord, the Black Sea and US Atlantic coast (Hargraves, 1990; Nanjappa *et al.*, 2014).

Some of the Chlorophyte strains genetically described in this study represent poorly characterized species. This was particularly the case for the strains of the genus *Picochlorum,* which clustered into two different clades, *Picochlorum* sp. 1 (AGSB-0012, AGSB-0118 and AGSB-0130) and *Picochlorum* sp. 2 (AGSB-0210). The genus *Picochlorum* is abundant in the world’s oceans, especially in temperate Atlantic waters near the US and European coasts, and is known to be a prevalent member of phytoplankton communities (Tragin and Vaulot, 2018). However, the precise identification of members of the genus *Picochlorum* is still challenging as they lack distinct morphological characteristics to distinguish between them, and sometimes from members of other genera. To date, the *Picochlorum* genus includes five species, four of which have been taxonomically accepted (Mucko *et al.*, 2020; Guiry and Guiry, 2024). An extended genetic comparison of those *Picochlorum* strains using additional marker genes such as the chloroplast 16S rRNA and rbcL should be considered for an accurate identification at the species (Mucko *et al.*, 2020).

Both of the *Chlamydomonas* strains identified in this study, AGSB-0055 and AGSB-0089 (both identified as *Chlamydomonas* spp.), form a sister clade to the psychrophile *C. priscuii* UWO241 (Pocock *et al.*, 2004; Stahl-Rommel *et al.*, 2022), which is a well-known Chlorophyte model system isolated from Lake Bonney (Antarctica) unable to grow at temperature above 18°C (Pocock *et al.*, 2004). Interestingly, UWO241 contains a highly similar insert in the same region of the 18S rRNA as the strain AGSB-0089. The other strain AGSB-0055 contains a different insert in the same region of the 18S rRNA. AGSB-0055, which was isolated from surface waters off the coast of Newfoundland, showed high similarity to *Cotesia euryale* previously isolated from coastal waters off the Lahave Island in Nova Scotia (Lewin, 1957).

### Limitations and perspective of long reads

Although there are still limitations using long-read sequencing techniques, including lower sequence accuracy of some systems, there is great potential to employ this approach for profiling phytoplankton communities from environmental samples. Nanopore sequencing technologies are reported to produce some amount of random base calling errors within reads; however, it has been demonstrated that generating consensus sequences from multiple sequence reads can correct minor inaccuracies (Kono and Arakawa, 2019; Davidov *et al.*, 2020; Sandin *et al.*, 2022). Most of the isolated strains characterized in this study were accurately identified with the 18S rRNA markers within 99% similarity, suggesting the use of consensus sequences overcame the accuracy limitation that might raise from nanopore’s high error rates which is reported to be higher than those of popular short-read sequencing platforms.

Combining information for the nearly full ribosomal genes through concatenated phylogenies results in a high-level of phylogenetic resolution, and provides long-read references needed to improve the taxonomic identification for future work on environmental phytoplankton communities. To date, the most common method used to describe phytoplankton communities consists of short-read metabarcoding of variable regions of the 18S rRNA. The V4 region within the 18S rRNA is the region of choice, as it has variable regions and the most comprehensive coverage in reference databases compared to other regions of the rRNA genes (Vaulot *et al.*, 2022). However, relying on this region alone can limit the diversity recovered from environmental samples. For example, when comparing the taxonomic resolution between short- and long-read sequencing for fungal species, Ohta and colleagues (Ohta *et al.*, 2023) demonstrated that long-read sequencing had superior discriminatory power, resulting in a more reliable and accurate taxonomic profiling. Comprehensive description of the prokaryotic communities using long reads are already well underway with pipelines using the entire 16S rRNA (Santos *et al.*, 2020; Matsuo *et al.*, 2021; Urban *et al.*, 2021). Using a similar approach for eukaryotic microbes, including phytoplankton, will likely benefit the description of those communities in the environment.

## CONCLUSIONS

This work not only illuminates the diversity of phytoplankton that can be isolated in the eastern Canadian waters, but also serves as a valuable resource for researchers globally, ultimately aiding the accurate taxonomic identification of phytoplankton. As shown in this study, the application of long-read sequencing technologies can simultaneously target different rRNA marker genes commonly used in taxonomic identification, connecting different reference databases available for microbial eukaryotes. This would significantly improve the characterization of environmental sequences, leading to an enhanced understanding of ecological functions within phytoplankton communities. Moreover, the information from large fragments of the ribosomal genes r, analyzed through concatenated phylogenies, results in a high level of phylogenetic resolution. This work provides the necessary long-read references required to improve taxonomic identification for future studies on environmental phytoplankton communities.

## Supplementary Material

Supplementary_Material_fbae043

## Data Availability

The rRNA sequences generated in this study were deposited in NCBI under the following accession numbers: OR922658 to OR922730.
