# Supplementary material for: Identification of phytoplankton isolates from the eastern Canadian waters using long-read sequencing
Source: J Plankton Res. 2024 Oct 3;46(6):527–41. doi: 10.1093/plankt/fbae043 (PMC11629783; doi:10.1093/plankt/fbae043)
Supplement: Supplementary_Material_fbae043 [file supplementary_material_fbae043.zip › Suppl_Figure1_2_fbae043.docx]

**Supplementary Tables**

**Identification of phytoplankton from** **Canadian Northwest Atlantic waters** **using long-read sequencing**

**Solenn Mordret**^1^**, Jenna MacKinnon**^1^**, Joerg Behnke**^1^**, Stephen J.B. O’Leary**^1^**, and Caroline Chénard**^1^*

^1^Aquatic and Crop Resource Development-National Research Council Canada, 1411 Oxford Street, Halifax, Nova Scotia, Canada B3H 3Z1

**Supplementary Table 1** - Primers used in this study. Each primer includes MinION adapters (5´-TTT CTG TTG GTG CTG ATA TTG C-forward primer-3´, 5´-ACT TGC CTG TCG CTC TAT CTT C-reverse primer-3´). Adapters are here displayed in green in the sequence.

| **Primer Name** | **FOR/REV** | **Sequence (5’ -> 3’)** | **Marker** | **References** |
| --- | --- | --- | --- | --- |
| **3NDF_Adapt** | F | TTT CTG TTG GTG CTG ATA TTG C GG CAA GTC TGG TGC CAG | 18S | Jamy et al, 2020 |
| **SSU-F_Adapt** | F | TTT CTG TTG GTG CTG ATA TTG C TC YAA GGA AGG CAG CAG GCG C | 18S | Hamsher et al., 2011 |
| **D3Ca-R_Adapt** | R | ACT TGC CTG TCG CTC TAT CTT C GA CGA SCG ATT TGC ACG TCA G | 28S | modified from Scholin et al., 1994a |
| **21R_Adapt** | R | ACT TGC CTG TCG CTC TAT CTT C GA CGA GGC ATT TGG CTA CCT T | 28S | Jamy et al, 2020 |

**Supplementary Table 2 -** Internal primers used to cut 18S and 28S rRNA

| **Primer Name** | **FOR/REV** | **Sequence (5’ -> 3’)** | **Marker** | **References** |
| --- | --- | --- | --- | --- |
| **1510F (V9)** | F | GTA GGT GAA CCT GCR GAA GG | 18S | Amaral-Zettler et al., 2009 |
| **D1R-C_m** | F | ACC CGC YGA AYT TAA GCA | 28S | Modified from Scholin et al., 1994b |

**Supplementary Table 3 -** Best BLAST Results for all sequences. BLASTn were conducted on Genbank (see link below). Length of full, 18S, ITS or 28S fragments is displayed including primers mentionned in Supp. Material 1 and 2. Best Blast for full sequences (full 18S, ITS1-5.8S-ITS2 , 28S rRNA fragment) was selected based on best "E value". Best Blast for 18S and 28S rRNA sequences were sorted based on best percent of identity (Id) . All query and id results are expressed in percentage.
