# Supplementary material for: Identification of phytoplankton isolates from the eastern Canadian waters using long-read sequencing
Source: J Plankton Res. 2024 Oct 3;46(6):527–41. doi: 10.1093/plankt/fbae043 (PMC11629783; doi:10.1093/plankt/fbae043)
Supplement: Supplementary_Material_fbae043 [file supplementary_material_fbae043.zip › Suppl_Material_rRNA_Nanopore_Manuscript_fbae043.docx]

**Supplementary Information**

**Identification of phytoplankton from** **Canadian Northwest Atlantic waters** **using long-read sequencing**

**Solenn Mordret**^1^**, Jenna MacKinnon**^1^**, Joerg Behnke**^1^**, Stephen J.B. O’Leary**^1^**, and Caroline Chénard**^1^*

^1^Aquatic and Crop Resource Development-National Research Council Canada, 1411 Oxford Street, Halifax, Nova Scotia, Canada B3H 3Z1


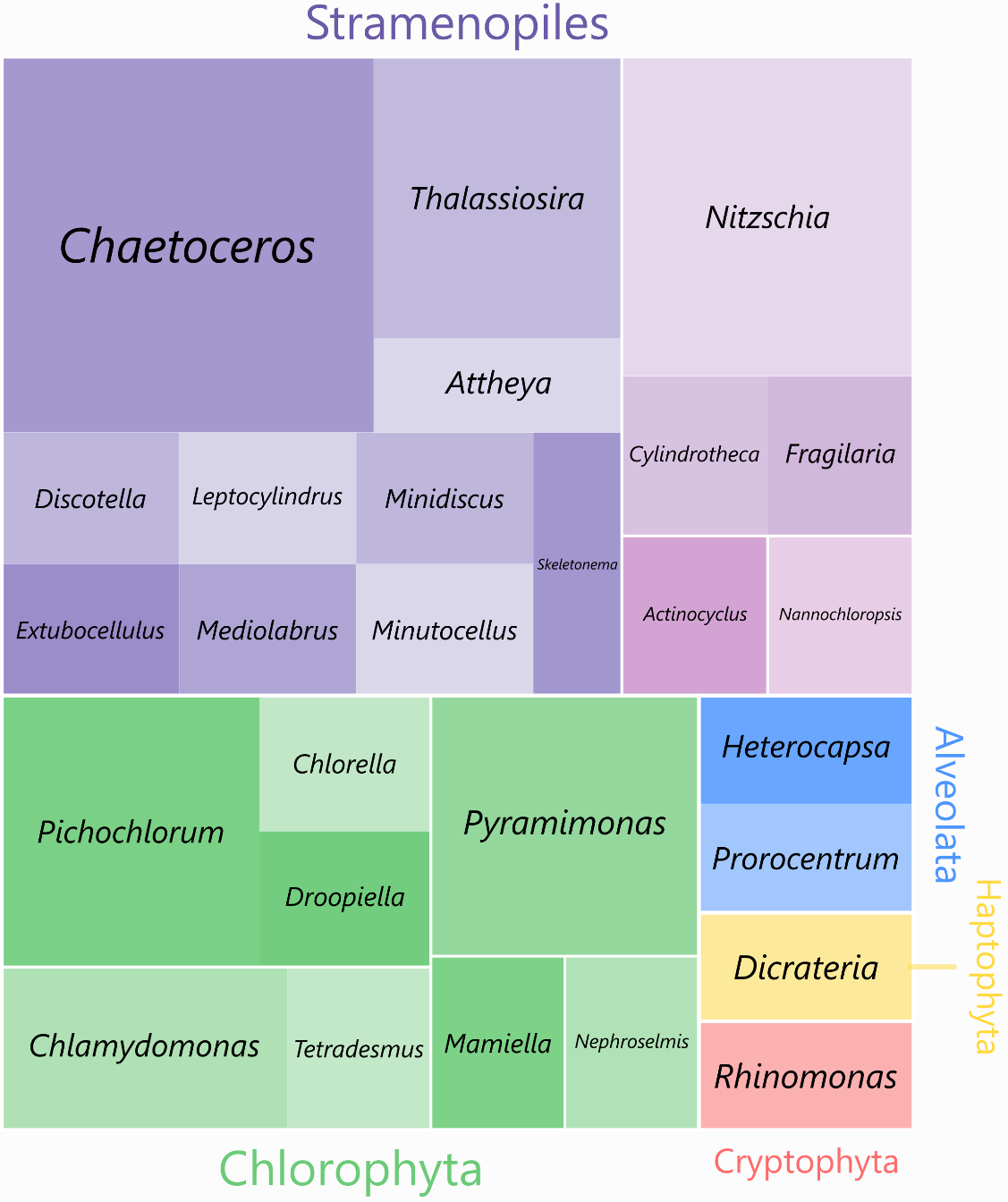


**Supplementary Figure 1-** Treemap showing overall diversity of phylotypes (division and genus level)

*
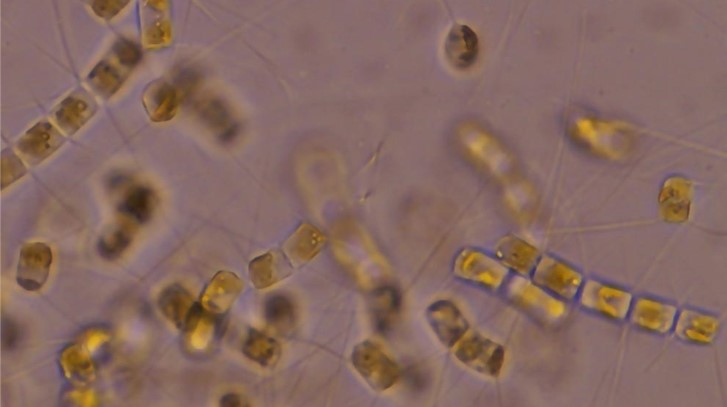
*

**Supplementary Figure 2** - Light Microscopy photograph of *Chaetoceros similis*. Cells measure around 7-10 µm.

**
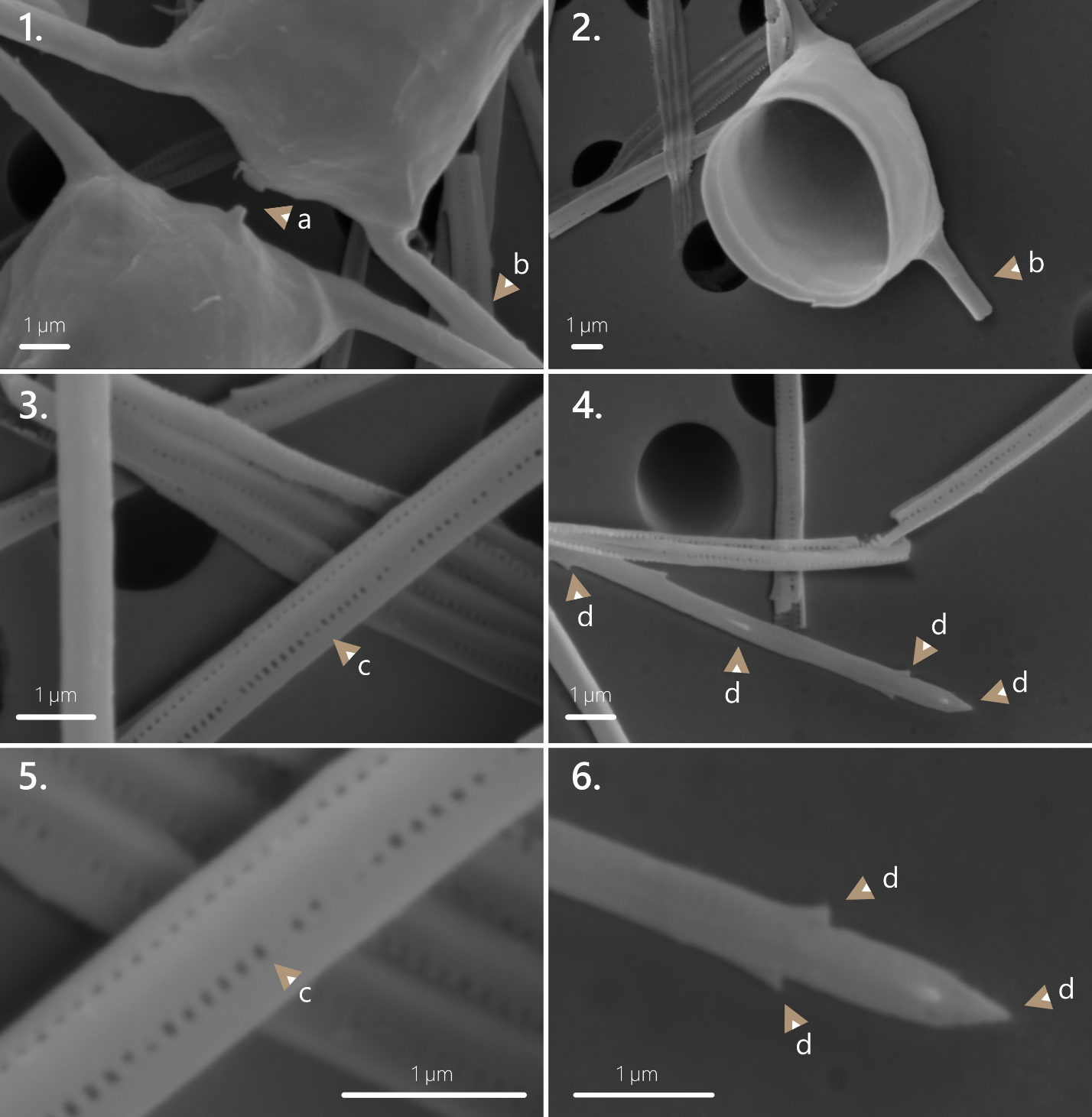
**

**Supplementary Figure 3**- SEM pictures of AGSB-0114 showing *Chaetoceros* *similis* morphological characterization which was used for taxonomic classification (1-6)*.* The arrows are showing the specific characteristics including the siblings’ valves with rimoportula (**a**), the setae that arise from the valves at an angle of 30-35° to the apical axis (**b**) and exhibit straight rows of slightly elongated vertically arranged poroids (**c**) on setae. Setae also display regular spines (**d**) arranged alternatively all around the setae. Scale bar = 1 µm.

**Material and Method – Supplementary Figure 3** -

AGSB-0114 strain culture was immersed in a fixative solution containing 2.5% (v/v) glutaraldehyde in 0.1 M of phosphate buffer and collected over Anodisc 25, Whatman, 0.2 µm filter membranes (Sigma-Aldrich) throughout the procedure. The samples were then dehydrated in 30%, 50%, 70%, 80%, and 90% ethanol (for 10 min each) and two times in 95% ethanol (20 min each) in succession at room temperature followed by HMDS (Supelco) treatment for 5 min at room temperature. The samples were then dried in a desiccator for 30 min and sputter-coated with gold for 120s using Cressington Model 108 Sputter Coater (Ted Pella) in order to prevent charging effects and improve the resolution. Scanning electron microscopy (SEM) analysis was performed on a Hitachi S-3000N SEM (Hitachi Scientific Instruments, Japan) using secondary electron detector operating in high vacuum mode at 0° angle, 20 kV accelerating voltage and a 5 mm working distance.
