# Supplementary material for: Identification of phytoplankton isolates from the eastern Canadian waters using long-read sequencing
Source: J Plankton Res. 2024 Oct 3;46(6):527–41. doi: 10.1093/plankt/fbae043 (PMC11629783; doi:10.1093/plankt/fbae043)
Supplement: Supplementary_Material_fbae043 [file supplementary_material_fbae043.zip › text_fbae043.pdf]

Strains IDs				Length and Location of 18S, 5.8S and 28S rRNA						
**Strains**	**GenBank Id**	**Species**	**Division**	**Full Seq length (bp)**	**18S rRNA length (bp)**	**18S location (bp)**	**5.8S rRNA length (bp)**	**5.8S rRNA location (bp)**	**28S rRNA length (bp)**	**28S location (bp)**
AGSB0001	OR922658	*Nitzschia* sp.	Stramenopiles	4240	1234	1-1234	154	1579-1733	2153	2088-4240
AGSB0002	OR922659	*Rhinomonas nottbecki*	Cryptophyta	4369	1216	1-1216	151	1598-1749	2320	2050-4369
AGSB0003	OR922660	*Attheya septentrionalis*	Stramenopiles	4632	1228	1-1228	153	1766-1919	2333	2300-4632
AGSB0004	OR922661	*Attheya septentrionalis*	Stramenopiles	4636	1229	1-1229	153	1768-1921	2334	2303-4636
AGSB0005	OR922662	*Attheya septentrionalis*	Stramenopiles	4617	1229	1-1229	153	1768-1921	2306	2312-4617
AGSB0006	OR922663	*Rhinomonas nottbecki*	Cryptophyta	4387	1216	1-1216	151	1599-1750	2337	2051-4387
AGSB0011	OR922664	*Tetradesmus obliquus*	Chlorophyta	4105	1219	1-1219	153	1456-1609	2228	1878-4105
AGSB0012	OR922665	*Picochlorum* sp.	Chlorophyta	4119	1227	1-1227	150	1397-1547	2228	1892-4119
AGSB0013	OR922666	*Prorocentrum pervagatum*	Alveolata	4272	1361	1-1361	154	1595-1749	2303	1970-4272
AGSB0014	OR922667	*Pyramimonas* sp.	Chlorophyta	4031	1223	1-1223	153	1411-1564	2243	1789-4031
AGSB0015	OR922668	*Chaetoceros socialis*	Stramenopiles	2958	1359	1-1359	154	1636-1790	865	2094-2958
AGSB0016	OR922669	*Nephroselmis pyriformis*	Chlorophyta	4523	1364	1-1364	151	1738-1889	2245	2279-4523
AGSB0017	OR922670	*Mediolabrus comicus*	Stramenopiles	4631	1214	1-1214	153	1481-1634	2714	1918-4631
AGSB0018	OR922671	*Minidiscus trioculatus*	Stramenopiles	4210	1217	1-1217	154	1483-1637	2280	1931-4210
AGSB0019	OR922672	*Nitzschia* sp.	Stramenopiles	4225	1220	1-1220	154	1483-1637	2164	2062-4225
AGSB0023	OR922673	*Pyramimonas obovata*	Chlorophyta	4101	1234	1-1234	153	1467-1620	2218	1884-4101
AGSB0025	OR922674	*Minutocellus polymorphus*	Stramenopiles	4391	1231	1-1231	154	1537-1691	2338	2054-4391
AGSB0026	OR922675	*Dicrateria rotunda*	Haptophyta	4302	1227	1-1227	153	1601-1754	2225	2078-4302
AGSB0027	OR922676	*Discostella* sp.	Stramenopiles	4201	1171	1-1171	154	1469-1623	2284	1918-4201
AGSB0030	OR922677	*Tetradesmus obliquus*	Chlorophyta	4621	1758	1-1758	151	1995-2146	2206	2416-4621
AGSB0031	OR922678	*Extubocellulus spinifer*	Stramenopiles	6497	2171	1-2171	154	3049-3203	2877	3621-6497
AGSB0032	OR922679	*Attheya septentrionalis*	Stramenopiles	4721	1228	1-1228	153	1903-2056	2284	2438-4721
AGSB0034	OR922680	*Thalassiosira hispida*	Stramenopiles	4363	1361	1-1361	154	1644-1798	2258	2106-4363
AGSB0037	OR922681	*Nannochloropsis granulata*	Stramenopiles	4579	1356	1-1356	154	1703-1857	2302	2278-4579
AGSB0038	OR922682	*Dicrateria rotunda*	Haptophyta	4405	1363	1-1363	154	1734-1888	2190	2216-4405
AGSB0040	OR922683	*Chlorella* sp.	Chlorophyta	3876	2307	1-2307	153	2604-2757	857	3020-3876
AGSB0041	OR922684	*Nitzschia* sp.	Stramenopiles	4272	1360	1-1360	154	1725-1879	2017	2256-4272
AGSB0044	OR922685	*Chaetoceros tenuissimus*	Stramenopiles	4300	1344	1-1344	152	1631-1783	2229	2072-4300
AGSB0045	OR922686	*Chaetoceros tenuissimus*	Stramenopiles	4314	1356	1-1356	153	1643-1796	2230	2085-4314
AGSB0049	OR922687	*Droopiella spaerckii*	Chlorophyta	4915	2568	1-2568	153	3253-3406	1302	3614-4915
AGSB0052	OR922688	*Chaetoceros tenuissimus*	Stramenopiles	4184	1221	1-1221	153	1508-1661	2235	1950-4184
AGSB0055	OR922689	*Chlamydomonas* sp.	Chlorophyta	4540	1219	1-1219	151	1508-1659	2652	1889-4540
AGSB0057	OR922690	*Thalassiosira aestivalis*	Stramenopiles	4640	1217	1-1217	154	1895-2049	2284	2357-4640
AGSB0058	OR922691	*Pyramimonas obovata*	Chlorophyta	4090	1227	1-1227	155	1461-1616	2212	1879-4090
AGSB0061	OR922692	*Heterocapsa steinii*	Alveolata	2993	1361	1-1361	154	1622-1776	984	2010-2993
AGSB0065	OR922693	*Leptocylindrus minimus*	Stramenopiles	4415	1365	1-1365	152	1700-1852	2222	2194-4415
AGSB0066	OR922694	*Cylindrotheca closterium*	Stramenopiles	4278	1352	1-1352	154	1618-1772	2160	2119-4278
AGSB0067	OR922695	*Skeletonema marinoi*	Stramenopiles	4696	1358	1-1358	153	1615-1768	2636	2061-4696
AGSB0068	OR922696	*Skeletonema marinoi*	Stramenopiles	4705	1360	1-1360	153	1617-1770	2643	2063-4705
AGSB0071	OR922697	*Chaetoceros socialis*	Stramenopiles	4323	1359	1-1359	154	1636-1790	2231	2093-4323
AGSB0082	OR922698	*Chaetoceros tenuissimus*	Stramenopiles	3878	1747	1-1747	153	2034-2187	1403	2476-3878
AGSB0085	OR922699	*Chaetoceros tenuissimus*	Stramenopiles	4696	1736	1-1736	152	2023-2175	2233	2464-4696
AGSB0087	OR922700	*Chaetoceros tenuissimus*	Stramenopiles	4288	1356	1-1356	152	1643-1795	2205	2084-4288
AGSB0088	OR922701	*Dicrateria rotunda*	Haptophyta	3031	1363	1-1363	154	1735-1889	809	2223-3031
AGSB0089	OR922702	*Chlamydomonas sp.*	Chlorophyta	5065	1767	1-1767	151	2461-2612	2223	2843-5065
AGSB0090	OR922703	*Extubocellulus spinifer*	Stramenopiles	5538	1770	1-1770	154	2086-2240	2879	2660-5538
AGSB0094	OR922704	*Chaetoceros neogracilis*	Stramenopiles	4285	1356	1-1356	154	1610-1764	2239	2047-4285
AGSB0096	OR922705	*Skeletonema marinoi*	Stramenopiles	4698	1357	1-1357	153	1614-1767	2639	2060-4698
AGSB0097	OR922706	*Nephroselmis pyriformis*	Chlorophyta	4521	1362	1-1362	153	1738-1891	2241	2281-4521
AGSB0102	OR922707	*Nitzschia* sp.	Stramenopiles	4937	1224	1-1224	153	1639-1792	2772	2166-4937
AGSB0111	OR922708	*Thalassiosira aestivalis*	Stramenopiles	4637	1227	1-1227	154	1904-2058	2273	2365-4637
AGSB0114	OR922709	*Chaetoceros similis*	Stramenopiles	4393	1359	1-1359	153	1709-1862	2215	2179-4393
AGSB0118	OR922710	*Picochlorum* sp.	Chlorophyta	4146	1227	1-1227	150	1397-1547	2248	1899-4146
AGSB0122	OR922711	*Chlamydomonas* sp.	Chlorophyta	4927	1633	1-1633	151	2325-2476	2221	2707-4927
AGSB0123	OR922712	*Chaetoceros tenuissimus*	Stramenopiles	4183	1221	1-1221	153	1508-1661	2234	1950-4183
AGSB0127	OR922713	*Dicrateria rotunda*	Haptophyta	4832	1767	1-1767	153	2141-2294	2204	2629-4832
AGSB0128	OR922714	*Picochlorum* sp.	Chlorophyta	4105	1219	1-1219	150	1389-1539	2224	1882-4105
AGSB0129	OR922715	*Thalassiosira aestivalis*	Stramenopiles	4754	1363	1-1363	154	2040-2194	2254	2501-4754
AGSB0130	OR922716	*Picochlorum* sp.	Chlorophyta	2881	1355	1-1355	150	1525-1675	862	2020-2881
AGSB0133	OR922717	*Cylindrotheca closterium*	Stramenopiles	4220	1352	1-1352	154	1581-1735	2166	2055-4220
AGSB0137	OR922718	*Chaetoceros tenuissimus*	Stramenopiles	4307	1355	1-1355	153	1642-1795	2224	2084-4307
AGSB0168	OR922719	*Thalassiosira pseudonana*	Stramenopiles	4347	1363	1-1363	154	1630-1784	2250	2098-4347
AGSB0176	OR922720	*Chaetoceros tenuissimus*	Stramenopiles	4668	1198	1-1198	153	1485-1638	2742	1927-4668
AGSB0181	OR922721	*Fragilaria famelica*	Stramenopiles	4878	1200	1-1200	154	1670-1824	2656	2223-4878
AGSB0186	OR922722	*Chaetoceros* sp.	Stramenopiles	5551	1355	1-1355	151	1647-1798	3436	2116-5551
AGSB0188	OR922723	*Thalassiosira pseudonana*	Stramenopiles	4725	1204	1-1204	154	1471-1625	2787	1936-4725
AGSB0210	OR922724	*Picochlorum* sp.	Chlorophyta	4822	1202	1-1202	153	1537-1690	2781	2042-4822
AGSB0221	OR922725	*Actinocyclus* sp.	Stramenopiles	5041	1361	1-1361	154	1852-2006	2637	2405-5041
AGSB0236	OR922726	*Droopiella spaerckii*	Chlorophyta	4965	2627	1-2627	153	3312-3465	1293	3673-4965
AGSB0242	OR922727	*Pyramimonas propulsa*	Chlorophyta	5614	2805	1-2805	153	2995-3148	2227	3388-5614
AGSB0244	OR922728	*Pyramimonas obovata*	Chlorophyta	4198	1355	1-1355	151	1589-1740	2196	2003-4198
AGSB0253	OR922729	*Picochlorum* sp.	Chlorophyta	2775	1351	1-1351	150	1521-1671	757	2019-2775
AGSB0254	OR922730	*Mamiella gilva*	Chlorophyta	4963	2256	1-2256	150	2427-2577	2114	2850-4963

	Best BLAST results											
**Strains**	**Best_Blast_full_seq**	**Best_Blast_fullseq_name**	**query_cover_fullseq**	**similarity_id_fullseq**	**Best_Blast_18S**	**Best_Blast_18S_name**	**query_cover_18S**	**similarity_id_18S**	**Best_Blast_28S**	**Best_Blast_28S_name**	**query_cover_28S**	**similarity_id_28S**
AGSB0001	OX595988	Nitzschia sp.	86	94.93	EU090031	Nitzschia sp.	100	99.19	OM891870	Nitzschia sp.	100	99.21
AGSB0002	MT796595	Rhodomonas baltica	96	93.02	LC647571	Rhinomonas nottbecki	100	99.92	HE820923	Rhodomonas salina	45	98.31
AGSB0003	OM891862	Stephanopyxis turis	81	87.33	LC189085	Attheya longicornis	100	100	MH020639	Attheya septentrionalis	39	99.78
AGSB0004	OM891862	Stephanopyxis turis	81	87.37	LC189085	Attheya longicornis	100	99.84	MH020639	Attheya septentrionalis	39	99.78
AGSB0005	OM891862	Stephanopyxis turis	81	87.31	LC189085	Attheya longicornis	100	99.84	MH020639	Attheya septentrionalis	39	99.78
AGSB0006	MT796595	Rhodomonas baltica	96	92.98	LC647571	Rhinomonas nottbecki	100	99.84	HE820923	Rhodomonas salina	45	98.12
AGSB0011	CP126209	Tetradesmus obliquus	100	99.81	MG971386	Tetradesmus obliquus	100	100	KC145458	Tetradesmus obliquus	81	99.78
AGSB0012	CP084613	Chorella sorokiniana	96	83.11	AB183583	Nannochloris sp.	100	99.76	AB437257	Chlorella variabilis	93	88.08
AGSB0013	JX402086	Prorocentrum minimum	100	97.94	OP094113	Prorocentrum pervagatum	100	100	AY822610	Prorocentrum donghaisense	100	98.35
AGSB0014	HE610154	Pyramimonas parkeae	70	91.88	JF794047	Pyramimonas sp. RCC2009	95	99.66	HE610154	Pyramimonas parkeae	100	95.34
AGSB0015	MW358650	Chaetoceros socialis	100	99.83	KY852276	Chaetoceros socialis	100	99.85	KY852296	Chaetoceros socialis	91	100
AGSB0016	HE610144	Nephroselmis pyriformis	59	99.7	AY425306	Nephroselmis pyriformis	99	100	HE610144	Nephroselmis pyriformis	99	99.87
AGSB0017	KY364698	Skeletonema costatum	89	92.19	MN528601	Mediolabrus comicus	95	99.91	LC258398	Skeletonema potamos	82	95.31
AGSB0018	KY364698	Skeletonema costatum	99	90.75	FJ590769	Minidiscus trioculatus	100	100	MT489355	Thalassiosira sp.	55	96.1
AGSB0019	OX595988	Nitzschia sp.	86	95.06	KY320383	Nitzschia paleaeformis	98	98.35	OM891870	Nitzschia traheaformis	99	97.05
AGSB0023	HE610152	Pyramimonas tetrarhynchus	70	88.2	KF422615	Pyramimonas obovata	97	99.83	HE610153	Pyramimonas olivacea	100	93.82
AGSB0025	KY364697	Cyclotella cryptica	86	86.11	MF001989	Minutocellus sp.	98	99.84	OM891871	Psammodictyon constrictum	93	90.63
AGSB0026	AF289038	Prymnesium patelliferum	61	93.31	LC519889	Dicrateria rotunda	100	100	AF289038	Prymnesium patelliferum	100	93.37
AGSB0027	KY364696	Cyclotella meneghiniana	99	86.61	DQ514902	Discotella sp.	98	92.04	AB831889	Discotella nipponica	50	97.25
AGSB0030	CP126209	Tetradesmus obliquus	100	99.74	MG022741	Tetradesmus obliquus	100	99.89	KC145458	Tetradesmus obliquus	82	99.78
AGSB0031	LC164794	Pseudoleyanella lunata	21	98.37	MF001983	Extubocellulus cupola	62	98.37	GQ219683	Leyanella arenaria	30	94.71
AGSB0032	OM891862	Stephanopyxis turis	82	87.11	LC189085	Attheya longicornis	100	100	MH020639	Attheya septentrionalis	40	99.78
AGSB0034	KY364697	Cyclotella cryptica	100	89.69	JN934691	Thalassiosira hispida RCC2521	96	99.92	MT489355	Thalassiosira sp.	55	97.93
AGSB0037	CP038111	Nannochloropsis oceanica	99	97.91	U41092	Nannochloropsis sp.	100	99.93	CP038132	Nannochloropsis oceanica	99	99.31
AGSB0038	AF289038	Prymnesium patelliferum	59	93.16	LC519889	Dicrateria rotunda	100	100	AF289040	Phaeocystis antarctica	94	93.41
AGSB0040	KY364701	Pseudochlorella pringsheimii	98	94.38	AY195981	Chlorella sp.	98	99.85	FR751203	Micratinium reisseri	96	97.71
AGSB0041	MT740317	Nitzschia anatoliensis	85	97.05	MN696723	Nitzschia cf. pusilla	97	98.19	OY101120	Nitzschia sp.	99	96.45
AGSB0044	MK331989	Chaetoceros tenuissimus	69	99.8	MG972315	Chaetoceros tenuissimus	100	99.93	MZ169567	Chaetoceros tenuissimus	40	99.89
AGSB0045	MK331989	Chaetoceros tenuissimus	69	99.77	MG972315	Chaetoceros tenuissimus	100	99.78	MZ169567	Chaetoceros tenuissimus	40	99.89
AGSB0049	MN248525	Droopiella spaerckii	79	99.56	KT860883	Chlorella stigmatophora	37	99.89	KJ871027	Oocystis sp.	63	96.11
AGSB0052	MK331989	Chaetoceros tenuissimus	68	99.68	MG972315	Chaetoceros tenuissimus	100	99.92	MZ169567	Chaetoceros tenuissimus	39	99.55
AGSB0055	KP313859	Chlamydomonas sp.	90	92.59	JF343798	Chlamydonas raudensis	94	99.22	LR798803	Chlamydomonas sp. CCMP235	83	97.24
AGSB0057	LC258398	Skeletonema potamos	49	96.23	AJ810857	Thalassiosira aestivalis	100	100	MT489355	Thalassiosira sp.	55	96.58
AGSB0058	HE610152	Pyramimonas tetrarhynchus	70	87.86	KF422615	Pyramimonas obovata	97	99.83	HE610153	Pyramimonas olivacea	100	93.59
AGSB0061	MF423347	Heterocapsa steinii	89	99.95	MF423350	Heterocapsa steinii	100	99.93	MF423357	Heterocapsa steinii	77	99.87
AGSB0065	AJ535176	Leptocylindrus minimum	31	99.28	KC309538	Leptocylindrus minimum	90	99.6	OM891885	Paralia sulcata	90	86.45
AGSB0066	AF289049	Cylindrotheca closterium	62	95.42	JF794039	Cylindrotheca closterium	95	99.69	AF289049	Cylindrotheca closterium	100	95.61
AGSB0067	KY364698	Skeletonema costatum	100	99.02	MN585960	Skeletonema marinoi	100	99.85	LC258382	Skeletonema marinoi	84	99.82
AGSB0068	KY364698	Skeletonema costatum	100	99.15	MN585960	Skeletonema marinoi	100	99.93	LC258381	Skeletonema marinoi	84	99.78
AGSB0071	JQ217339	Chaetoceros socialis	76	99.67	KY852276	Chaetoceros socialis	100	99.85	MW358650	Chaetoceros socialis	41	99.89
AGSB0082	MK331989	Chaetoceros tenuissimus	86	97.7	KM401854	Chaetoceros dayaensis	91	96.28	MK193876	Chaetoceros tenuissimus	41	99.83
AGSB0085	MK331989	Chaetoceros tenuissimus	71	99.73	KY852257	Chaetoceros tenuissimus	100	99.94	MZ169567	Chaetoceros tenuissimus	39	99.78
AGSB0087	MK331989	Chaetoceros tenuissimus	69	99.56	MG972315	Chaetoceros tenuissimus	100	100	MG914624	Chaetoceros tenuissimus	37	99.76
AGSB0088	LC519889	Dicrateria rotunda	45	100	LC519889	Dicrateria rotunda	100	100	EU729457	Dicrateria rotunda	84	99.27
AGSB0089	KP313859	Chlamydomonas sp.	83	93.09	JF343798	Chlamydomonas raudensis	73	99.87	KP313859	Chlamydomonas sp.	99	96.31
AGSB0090	FR865495	Extubocellulus sp.	40	99.24	KT861092	Extubocellulus sp. RCC975	38	99.71	GQ219683	Leyanella arenaria	30	94.83
AGSB0094	JQ217339	Chaetoceros socialis	75	86.36	EU090014	Chaetoceros sp.	99	99.93	MZ267683	Chaetoceros costatus	100	91.88
AGSB0096	KY364698	Skeletonema costatum	100	99.09	MN585960	Skeletonema marinoi	100	99.78	LC258382	Skeletonema marinoi	84	99.87
AGSB0097	HE610144	Nephroselmis pyriformis	59	99.65	AY425306	Nephroselmis pyriformis	100	99.93	HE610144	Nephroselmis pyriformis	100	99.82
AGSB0102	OX595990	Nitzschia sp.	87	96.11	MN750472	Nitzschia sp.	99	100	OY101120	Nitzschia sp.	100	97.08
AGSB0111	LC258398	Skeletonema potamos	59	88.83	AJ810857	Thalassiosira aestivalis	100	99.84	MT489355	Thalassiosira sp.	55	96.58
AGSB0114	MZ267686	Chaetoceros laevisporus	50	91.65	LC189089	Chaetoceros sp. NIES3971	100	99.78	MZ267686	Chaetoceros laevisporus	99	91.65
AGSB0118	AB237642	Chlorella vulgaris	54	86.96	AB183583	Nannochloris sp.	100	99.76	AB437257	Chlorella variabilis	92	87.98
AGSB0122	KP313859	Chlamydomonas sp.	82	93.02	KT860823	Chlamydomonas sp. RCC2607	39	100	LR798803	Chlamydomonas sp. CCMP235	99	96.27
AGSB0123	MK331989	Chaetoceros tenuissimus	68	99.75	MG972315	Chaetoceros tenuissimus	100	99.84	MZ169567	Chaetoceros tenuissimus	39	99.89
AGSB0127	LC519889	Dicrateria rotunda	36	99.94	LC519889	Dicrateria rotunda	100	99.94	AF289038	Prymnesium patelliferum	100	93.08
AGSB0128	CP084613	Chorella sorokiniana	96	83.19	AB183583	Nannochloris sp.	100	99.84	AB437257	Chlorella variabilis	93	88.14
AGSB0129	LC258398	Skeletonema potamos	57	96.05	AJ810857	Thalassiosira aestivalis	100	99.93	MT489355	Thalassiosira sp.	55	96.58
AGSB0130	AB183583	Nannochloris sp.	47	99.85	AB183583	Nannochloris sp.	100	99.85	HF536547	Micratinium reisseri	100	86.36
AGSB0133	MT489381	Cylindrotheca sp.	76	97.37	HQ912645	Cylindrotheca closterium	98	99.93	AF289049	Cylindrotheca closterium	100	99.31
AGSB0137	MK331989	Chaetoceros tenuissimus	69	99.73	MG972315	Chaetoceros tenuissimus	100	99.93	MZ169567	Chaetoceros tenuissimus	40	99.78
AGSB0168	KY364697	Cyclotella cryptica	100	91.84	MT383673	Thalassiosira pseudonana	100	100	XM_002294498	Thalassiosira pseudonana	73	100
AGSB0176	MK331989	Chaetoceros tenuissimus	60	99.75	MG972315	Chaetoceros tenuissimus	100	99.92	MZ267685	Chaetoceros tenuissimus	100	97.47
AGSB0181	OY101118	Nitzschia sp.	84	91.27	EF423415	Synedra minuscula	100	99.92	OY101120	Nitzschia sp.	100	91.45
AGSB0186	MZ267683	Chaetoceros costatus	40	87	DQ830988	Chaetoceros sp. Jonquieri	100	100	OM891869	Cylindrotheca closterium	86	88.63
AGSB0188	KY364697	Cyclotella cryptica	100	92.32	MT383673	Thalassiosira pseudonana	100	99.92	XM_002294498	Thalassiosira pseudonana	59	99.94
AGSB0210	AB237642	Chlorella vulgaris	58	92.18	AY422075	Picochlorum oculatum	97	99.49	AB237642	Chlorella vulgaris	100	92.19
AGSB0221	X85401	Actinocyclus curvatulus	35	99.06	OP304750	Actinocylcus sp.	90	99.51	XM_002294498	Thalassiosira pseudonana	53	78.63
AGSB0236	MN248525	Droopiella spaerckii	78	99.56	KT860883	Chlorella stigmatophora	36	99.89	KJ871027	Oocystis sp.	63	95.5
AGSB0242	HE610152	Pyramimonas tetrarhynchus	74	93.55	AB017123	Pyramimonas propulsa	48	99.78	AB491638	Pyramimonas parkeae	98	99.5
AGSB0244	HE610152	Pyramimonas tetrarhynchus	68	88.29	KF422615	Pyramimonas obovata	97	100	HE610153	Pyramimonas olivacea	100	94.12
AGSB0253	AB183583	Nannochloris sp.	48	99.93	AB183583	Nannochloris sp.	100	99.93	FR850476	Coccomyxa sp.	56	87.56
AGSB0254	CP001575	Micromonas sp. RCC299	83	92.52	FN562450	Mamiella gilva	70	99.71	HE610150	Mantoniella squamata	100	95.61
